# Characteristics of Chronic Pain among Head and Neck Cancer Patients Treated with Radiation Therapy: A Retrospective Study

**DOI:** 10.1155/2019/9675654

**Published:** 2019-05-06

**Authors:** Anusha Kallurkar, Shreedhar Kulkarni, Kristin Delfino, Daniel Ferraro, Krishna Rao

**Affiliations:** ^1^Department of Anesthesiology, Louisiana State University Health Sciences Center at Shreveport, Shreveport, LA, USA; ^2^Division of Medicine/Psychiatry, Department of Medicine, SIU School of Medicine, Springfield, IL, USA; ^3^Department of Surgery, SIU School of Medicine, Springfield, IL, USA; ^4^University Radiologists, Memorial Medical Center, Springfield, IL, USA; ^5^Division of Hematology/Oncology, Simmons Cancer Institute at SIU School of Medicine, Springfield, IL, USA

## Abstract

Pain is common among patients with head and neck cancer (HNC). However, there are very limited data on chronic pain among HNC patients treated with radiation therapy (XRT). In this retrospective study, we focused on the characteristics of chronic post-XRT pain in such patients. Post-XRT pain is common among HNC patients; however, we found discrepancy between frequency of treatment and frequency of chronic pain, suggesting poor documentation of pain in the medical records. Among patients who reported to have chronic post-XRT pain, most of them described having severe pain and used descriptors of neuropathic pain. Pharynx was the commonest site of cancer as well as the commonest site of cancer-related chronic pain; squamous cell carcinoma was the most frequent histological pattern, and opioids were used most often to treat such chronic pain. There was a significant association between chronic pain and number of sites of pain, and chronic pain was also associated with use of opioids.

## 1. Introduction

Pain is common among patients with head and neck cancer (HNC) prior to treatment and may be attributed to the cancer and/or cancer treatment. Pain in HNC patients could be due to tissue damage from several mechanisms such as mucosal injury, nerve compression, and invasion of the tumor into adjacent tissue structures with inflammation or ischemia [1]. Management of HNC includes stage-dependent single or multimodality approaches comprising surgery, chemotherapy, and/or radiation therapy (XRT). Postoperative pain can be due to tissue injury with muscle spasm and nerve injury leading to a combination of inflammatory and neuropathic components [2]. Chemotherapy-induced peripheral neuropathic pain is common and can be due to a combination of mitochondrial dysfunction, changes in expression of various cytokines, and abnormal spontaneous discharge in A and C fibers [3]. XRT can induce oral mucositis in patients with HNC and can cause oral pain due to impaired wound healing [4]. Thus, all of these modalities of treatment can cause acute pain, which often resolves within the critical three-month time span after treatment.

HNC can take up to 3 months to disappear histologically after completion of treatment, thus indicating remission of disease [5]. The follow-up visit at 3 months of completion of cancer therapy is particularly important since it helps in establishing the baseline result of treatment response and also marks the timing of the posttreatment baseline imaging [6, 7]. A negative PET-CT scan after treatment with chemoradiotherapy in HNC is associated with a high negative predictive value (>95%), and a negative scan around 3 months after completion of cancer therapy may indicate no residual disease [5, 8]. Patients who achieve a complete remission at the three-month posttreatment time point and who are still experiencing pain are thought to be having chronic pain. According to the International Association for the Study of Pain (IASP), “chronic pain” is defined as “the pain that has persisted beyond normal tissue healing time,” lasting for “more than 3 months” in the absence of other criteria [9].

Oral mucositis is a common cause of acute pain in HNC patients, and it typically lasts for 2–4 weeks after the end of radiation therapy (XRT) [10]. However, HNC patients can experience pain even up to 6–12 months after the radiation therapy [11]. There are very limited data on the prevalence and characteristics of chronic pain among HNC patients treated with XRT, and it is not clear which subgroup of HNC patients is more prone for chronic pain after XRT. In this retrospective observational study, we investigated the prevalence of chronic pain and the characteristics of patients in complete remission afflicted with chronic posttreatment pain.

## 2. Methods

### 2.1. Design

This retrospective study was conducted at Southern Illinois University (SIU) School of Medicine and Memorial Medical Center, Springfield, IL. All head and neck cancer patients aged 18 years and above, treated with radiotherapy from February 2011 to February 2016, were included in the study. Head and neck cancer patients treated only with chemotherapy were excluded. The chart review identified a total of 53 patients, who met the inclusion criteria. Demographic features such as age at diagnosis, gender and race, clinicopathologic features (location, histology, and staging of the cancer), cancer therapy (surgery and chemotherapy) and details pertinent to XRT (such as dose, duration, and area involved) were collected. In terms of location, data pertaining to upper aerodigestive tract sites (oral cavity, pharynx, larynx, nasal cavity, and paranasal sinuses) as well as salivary glands were collected. We focused on cancers of the oral cavity, pharynx, and larynx in this study because of their similarities in epidemiology, treatment, and prognosis; cancers of the lip, salivary gland, nose, paranasal sinuses, middle ear, nerves and bones, thyroid, nonmelanoma skin cancers, lymphoma, and sarcomas were excluded.

Data were also collected regarding the characteristics of pain: location, onset, frequency, severity, nature of pain, and information regarding use of medications including opioids, gabapentin, TCAs, and NSAIDs. Data regarding characteristics of pain up to 3 months after completion of XRT were categorized as “data related to acute pain,” and subsequent data (after 3 months of completion of XRT) were considered as “data related to chronic pain.” Pain was documented as mild, moderate, or severe based on the documentation in the charts. If pain severity was documented numerically, it was converted into mild (documented as 1–3 in the charts), moderate [4–6], and severe [7–10]. The local institutional review board approved the study, protocol number 17-021 (approval date 02/20/2017).

### 2.2. Statistical Analyses

Data were analyzed with the use of SAS software, version 9.4, and Zotero bibliography software was used for citing references. Descriptive statistics were computed for all study variables. Continuous variables are described with measures of central tendency (mean and median) and dispersion (range and standard deviation). Categorical variables are summarized as frequencies and percentages. Given smaller cell sizes, the following adaptations were made during statistical analyses: cancer stages were grouped into “stage 1 or 2” and “stage 3 or 4”; data for mild, moderate, and severe pain were combined into one group, and number of sites of pain were grouped into three categories such as “pain at 0 site,” “pain at 1 site,” and “pain at 2 + sites.” Statistical analysis was not performed for use of TCA's given smaller sample size. Fisher's exact tests were used to compare categorical variables. Independent *t*-tests (or nonparametric equivalent) were used to compare continuous variables. All significance was assumed at the *p* < 0.05 level.

## 3. Results

Demographic and clinicopathologic factors are described in Tables 1 (categorical variables) and 2 (continuous variables). Mean age at diagnosis was 61.23 years, and 73.6% were males. 88.7% of the patients had squamous cell carcinoma (*n* = 47), and other types of histology included adenocarcinoma (*n* = 1), high-grade neuroendocrine tumor (*n* = 1), solitary extramedullary plasmacytoma (*n* = 1), and adenoid cystic carcinoma (*n* = 1).

A total of 41 patients (77.4%) reported any kind of treatment for chronic pain, whereas only 32 patients (60.38%) reported any kind of chronic pain, suggesting poor documentation of pain characteristics in the charts. There was insufficient description of nature of pain; among the patients who reported any kind of chronic pain (*n* = 32), only 4 individuals described the nature of their pain (all of them used descriptors of neuropathic pain such as stinging or burning pain) and less than 4 individuals reported onset, frequency of chronic pain, and exacerbating features. Eleven patients died during the study period with average duration of survival of 15 months.

Pharynx (Table 3) was the most common location of cancer (39.6%), and oropharynx was the most frequent subsite of HNC (34%). Site of cancer was associated with use of medications. Specifically, laryngeal cancer (*p*=0.01; supraglottic cancer, *p*=0.021) was associated with higher use of gabapentin, and cancer of pharynx (*p*=0.016; cancer of oropharynx, *p*=0.04) was associated with lower use of gabapentin.

Pharynx (26.4%) was the commonest site of cancer-related chronic pain, followed by oral cavity (24.5%) (Table 4). Chronic neck pain was associated with use of gabapentin (*p*=0.013).

There was a significant association between chronic pain and number of pain sites (*p* < 0.0001), and chronic pain was also associated with use of opioids (*p*=0.006) (Table 5). There was no significant association between site of cancer and site of cancer-related chronic pain. Surgery was not associated with chronic pain or use of pain medications (details of statistical analyses for surgery, chemotherapy, XRT dose, and duration have been listed as separate tables, which can be found in Tables 6–9, respectively). Similarly, chemotherapy had no effect on chronic pain or use of analgesics. XRT dose had no effect on chronic pain or use of pain medications. Likewise, XRT duration was not associated with chronic pain or use of analgesics.

## 4. Discussion

Pain is common among HNC patients and can be nociceptive, inflammatory, or neuropathic in nature. Nociceptive pain refers to the response to noxious stimuli and continues in the maintained presence of noxious stimuli [12]. Although inflammatory pain typically improves with resolution of inflammation, there is growing evidence to support that in some cases inflammatory state may resolve but a component of pain persists [13]. In an animal model, Christianson et al. showed that chronic inflammatory states may mimic neuropathic-like pain and continue to have persistent allodynia despite resolution of inflammation, and such allodynia responded only to gabapentin [14]. Pathophysiological changes in the pain pathways resulting from repetitive nociceptive stimulation lead to peripheral or central sensitization, which ultimately leads to transition of acute pain into chronic pain in susceptible individuals [15].

Pain is common among patients with HNC; acute post-XRT pain typically lasts for less than 3 months whereas chronic pain lasts for more than 3 months. Based on our clinical experience in managing post-XRT patients with HNC, we observed that chronic pain is commonly encountered; however, there are very limited data in the literature on the characteristics of chronic pain among HNC patients treated with XRT. Kuo and Williams listed 14 studies investigating pain in HNC patients, in which 3 studies reported persistence of pain up to 6–24 months, and prevalence of pain varied from 15% to 46% [16]. Only one study focused on post-XRT pain in HNC patients [11]. Recently, Srivastava et al. reported 54.7% patients reporting chronic post-XRT pain [17]. In our study, the sample size is larger, and we found almost two-thirds of patients to have some characteristics of chronic pain. The higher rate of prevalence of chronic pain in our study could be due to methodological differences as we included any documentation pertaining to chronic pain (location, nature, frequency of pain, etc.) in the study analysis.

We found discrepancy between frequency of treatment and frequency of chronic pain suggesting poor documentation of pain in the medical records, and less than 4 individuals reported onset (*n* = 1), frequency (*n* = 3), and exacerbating factors (*n* = 2) of chronic pain. It is difficult to generalize these data, given small proportion of patients receiving appropriate documentation. Nevertheless, this phenomenon of inadequate pain documentation is not limited to patients with HNC. Fink [18] reported poor documentation of acute and chronic noncancer pain. However, appropriate cancer pain documentation is one of the key elements of the American Society of Clinical Oncology (ASCO) Quality Oncology Practice Initiative (QOPI), and Ranpura et al. showed improvement in documentation of pain among cancer patients with orientation and education of healthcare providers [19].

Mean age at diagnosis in our group was 61.23 years [20], and almost three-fourths of the patients were males (*n* = 39), which is comparable to other studies in the literature [21]. However, we found that most of our patients were Caucasians (*n* = 46, 86.8%) although historically, HNC is more common in African American individuals; this statistic probably reflects local demographics of Central Illinois. According to a population survey in 2013 [22], average Caucasian population in this area is 90.45%. Also, HPV-positive cases of HNC are more common in Caucasians, and we did not assess for HPV status in our study.

Among 53 patients in our study, pharynx was the most common site (39.6%), followed by larynx (32.1%), and among subsites of larynx, supraglottic carcinoma (17%) was the most common site that is consistent with the previous literature [23, 24]. In our study, laryngeal cancer (supraglottic cancer in particular) was associated with higher use of gabapentin. Supraglottic cancers usually have worse prognosis, given high frequency of cervical lymph node involvement, recurrences, and visceral metastases, likely explaining the increased use of analgesics.

Site of chronic pain was also associated with use of medications; chronic neck pain was associated with higher use of gabapentin. HNC patients treated with XRT are found to have neuropathic pain [25], and gabapentin is well established to be effective in treating neuropathic pain and seems to be promising in reducing the need for higher doses of opioids [26]. Gabapentin and opioids are commonly prescribed for pain, the likelihood of coprescription is high, and gabapentin appears to potentiate the effect of opioids [27].

There was a significant association between chronic pain and number of pain sites, and individuals with chronic pain were more likely to be treated with opioids. Murphy et al. found increased pain in HNC population to be associated with increased use of opioids [28]. Epstein et al. showed HNC patients treated with XRT to have both nociceptive and neuropathic pain despite ongoing pain management during XRT [25]. Chronic pain has been attributed to radiation therapy among patients with other cancers such as cervical cancer and breast cancer [29–31]. Several explanations are possible, explaining the chronic nature of pain among HNC patients treated with XRT. It could be related to chronic neuropathic pain or secondary to radiation-induced fibrosis as well as due to effects of XRT on the lymphatic system. Peuckmann et al. found almost half of the patients with chronic in pain breast cancer patients to have paresthesia of the skin corresponding to the areas of surgery and XRT [31]. Allodynia was associated with XRT only. Another mechanism could be that the irradiated tissue developing increased vascular permeability which in turn leads to fibrin deposition, subsequent collagen formation, and fibrosis. Such radiation-induced fibrosis could damage peripheral nerves which could lead to chronic neuropathic pain [32–34]. Thus, it is possible that such neuropathic pain secondary to XRT might be contributing to chronic pain among HNC patients treated with XRT. XRT could cause somatic pain related to osteonecrosis of bone, and it could also disrupt lymphatics, causing lymphedema and chronic swelling and likely inflammatory pain [35]. However, further research is needed in this field with prospective studies evaluating nature of chronic pain and risk factors among HNC patients treated with XRT.

Higher doses of XRT are known to have a positive correlation with chronic pain among HNC patients, possibly related to radiation-induced fibroatrophic processes [36]. However, we did not find any significant association of XRT dose or XRT duration with chronic pain or with use of pain medications (Tables 8 and 9). This could be due to the lower than average XRT dose used in our study. The average XRT dose in our study was 6063.34 cGy, which is lower as compared to the standard regimen of 7000 cGy. The lower dose may be due to refusal of further XRT by patients due to side effects of treatment and overall poor quality of life.

In our study group, surgery was not associated with chronic pain or use of pain medications (Table 6). Similarly, chemotherapy had no effect on chronic pain or use of analgesics (Table 7). There was no difference in rates of survival among patients with chronic pain as compared to those without pain. We did not have any data on use of botulinum toxin injection in HNC patients. Type-A botulinum toxin is an analgesic and a muscle relaxant and has been used to treat pain related to neck muscle spasm and contracture in post-XRT HNC patients. Along with conventional analgesics, future studies should explore Botulinum toxin injection and other therapies as therapeutic options to treat chronic pain in post-XRT HNC patients [37, 38].

Limitations of our study include the fact that it is a retrospective single center study. Comorbid chronic medical conditions were also not adjusted during the statistical analysis. The overall sample size in our study is small, and particularly, very small cell sizes (*n* < 8) should be interpreted with caution.

## 5. Conclusions

In summary, post-XRT pain among HNC patients is common not only during the acute period but also in the chronic period lasting well beyond three months after completion of XRT. We found discrepancy between frequency of treatment and frequency of chronic pain, suggesting poor documentation of pain in the medical records. Among patients who reported having chronic post-XRT pain, most of them described having severe pain and used descriptors of neuropathic pain. Pharynx was the commonest site of cancer as well as the commonest site of cancer-related chronic pain; squamous cell carcinoma was the most frequent histological pattern, and opioids were used most often to treat such chronic pain. There was a significant association between chronic pain and number of pain sites, and chronic pain was associated with use of opioids. Surgery was not associated with chronic pain or use of pain medications. Similarly, chemotherapy had no effect on chronic pain or use of analgesics. XRT dose had no effect on chronic pain or use of pain medications. Likewise, XRT duration was not associated with chronic pain or use of pain medications. There was no difference in rates of survival among patients with chronic pain as compared to those without pain. Site of cancer and site of cancer-related chronic pain were associated with use of pain medications. However, these results have to be carefully interpreted, given small sample size. Further research using prospective studies with larger samples is needed to explore the characteristics of chronic pain among HNC patients treated with XRT and parse the roles of chemotherapy and radiation.

## Figures and Tables

**Table 1:** Demographic and clinicopathologic factors (categorical variables) (frequency and percentage of demographic factors including age, gender, and race and clinicopathologic factors such as histology, cancer staging, severity of pain, and cancer treatment).

Variable		*n*	%
*Categorical variables*			
Gender	Female	14	26.4
	Male	39	73.6

Race	White	46	86.8
	AA	5	9.4

Histology	Squamous	47	88.7

Staging	1	4	7.6
	2	4	7.6
	3	5	9.4
	4	21	39.6

Surgery	No	32	60.4
	Yes	21	39.6

Chemo	No	21	39.6
	Yes	32	60.4

Severity of chronic pain	No pain	35	66.0
	Mild	1	1.9
	Moderate	4	7.5
	Severe	13	24.5

*Medication*			
Use of opioids	No	20	37.7
	Yes	33	62.3

Use of gabapentin	No	45	84.9
	Yes	8	15.1

Use of TCAs for chronic pain	No	47	88.7
	Yes	6	11.3

Use of NSAIDs	No	39	73.6
	Yes	14	26.4

**Table 2:** Demographic and clinicopathologic factors (continuous variables).

Variable	*n*	Mean	Std. error	Median	Std. dev.	Min.	Max.
Age at diagnosis	53	61.23	1.73	59.00	12.62	40.00	91.00
XRT dose	53	6063.34	201.30	6600.00	1465.47	600.00	7000.00
XRT duration	53	43.04	1.77	45.00	12.85	4.00	75.00
Number of sites of pain	53.00	1.21	0.19	1.00	1.38	0.00	6.00

**Table 3:** Site of cancer: frequency and percentage of prevalence of cancer by site and subsites and association between site of cancer and use of medications^*∗*^.

Site of cancer		Total		Use of opioids		Use of NSAIDs		Use of gabapentin	
		*n*	%	% yes	*p*	% yes	*p*	% yes	*p*
**1. Oral cavity**	No	47	88.7	61.7	1	25.5	0.649	12.8	0.219
	Yes	6	11.3	66.7		33.3		33.3	

1A. Lip	No	53	100	62.3	—	26.4	—	15.1	—
	Yes	0	0						

1B. Buccal mucosa	No	53	100	62.3	—	26.4	—	15.1	—
	Yes	0	0						

1C. Alveolar ridge, retromolar trigone	No	50	94.3	62.0	1	24.0	0.167	16.0	1
	Yes	3	5.7	66.7		66.7		0.0	

1D. Floor of mouth	No	50	94.3	60.0	0.282	26.0	1	12.0	0.056
	Yes	3	5.7	100.0		33.3		66.7	

1E. Hard palate	No	52	98.1	63.5	0.377	26.9	1	15.4	1
	Yes	1	1.9	0.0		0.0		0.0	

1F. Oral tongue	No	51	96.2	60.8	0.521	25.5	0.462	15.7	1
	Yes	2	3.8	100.0		50.0		0.0	

**2. Pharynx**	No	32	60.4	62.5	1	31.3	0.362	25.0	**0.016**
	Yes	21	39.6	61.9		19.0		0.0	

2A. Nasopharynx	No	52	98.1	63.5	0.377	26.9	1	15.4	1
	Yes	1	1.9	0.0		0.0		0.0	

**2B. Oropharynx**	No	35	66	60.0	0.768	25.7	1	22.9	**0.04**
	Yes	18	34	66.7		27.8		0.0	

**2C. Oropharynx: base of tongue**	No	44	83	63.6	0.715	27.3	1	18.2	0.324
	Yes	9	17	55.6		22.2		0.0	

2D. Soft palate	No	49	92.5	63.3	0.627	28.6	0.563	16.3	1
	Yes	4	7.5	50.0		0.0		0.0	

**2E. Tonsillar fossa pillar**	No	43	81.1	58.1	0.286	25.6	1	18.6	0.327
	Yes	10	18.9	80.0		30.0		0.0	

**2F. Hypopharynx, pyriform sinus**	No	46	86.8	65.2	0.405	30.4	0.17	17.4	0.577
	Yes	7	13.2	42.9		0.0		0.0	

**3. Larynx**	No	36	67.9	55.6	0.225	19.4	0.109	5.6	**0.01**
	Yes	17	32.1	76.5		41.2		35.3	

**3A. Supraglottis**	No	44	83	56.8	0.129	27.3	1	9.1	**0.021**
	Yes	9	17	88.9		22.2		44.4	

**3B. Glottis**	No	46	86.8	60.9	0.697	21.7	0.07	13.0	0.283
	Yes	7	13.2	71.4		57.1		28.6	

3C. Subglottis	No	50	94.3	60.0	0.282	26.0	1	14.0	0.394
	Yes	3	5.7	100.0		33.3		33.3	

4. Nasal cavity	No	50	94.3	62.0	1	28.0	0.557	16.0	1
	Yes	3	5.7	66.7		0.0		0.0	

5. Paranasal sinuses	No	53	100	62.3	—	26.4	—	15.1	—
	Yes	0	0						

6. Others	No	50	94.3	64.0	0.549	28.0	0.557	16.0	1
	Yes	3	5.7	33.3		0.0		0.0	

7. Salivary gland	No	50	94.3	66.0	0.049	26.0	1	16.0	1
	Yes	3	5.7	0.0		33.3		0.0	

^*∗*^Several cell sizes are very small and have been excluded from final statistical analyses. The ones that are included in the interpretation of final results are described in bold text.

**Table 4:** Various sites of pain among HNC patients and association between site of pain and use of medications^*∗*^.

Site of pain		Total		Use of opioids		Use of NSAIDs		Use of gabapentin	
		*n*	%	% yes	*p*	% yes	*p*	% yes	*p*
**1. Oral cavity**	No	40	75.5	55.0	0.098	27.5	1	15.0	1
	Yes	13	24.5	84.6		23.1		15.4	

**2. Pharynx**	No	39	73.6	56.4	0.203	28.2	0.735	17.9	0.665
	Yes	14	26.4	78.6		21.4		7.1	

3. Eyes	No	52	98.1	61.5	1	26.9	1	15.4	1
	Yes	1	1.9	100.0		0.0		0.0	

4. Nasal cavity, paranasal sinuses	No	52	98.1	61.5	1	26.9	1	15.4	1
	Yes	1	1.9	100.0		0.0		0.0	

**5. Odynophagia**	No	44	83	59.1	0.456	25.0	0.684	13.6	0.611
	Yes	9	17	77.8		33.3		22.2	

6. Headache	No	50	94.3	60.0	0.282	24.0	0.167	14.0	0.394
	Yes	3	5.7	100.0		66.7		33.3	

**7. Face**	No	48	90.6	58.3	0.144	27.1	1	14.6	0.574
	Yes	5	9.4	100.0		20.0		20.0	

**8. Ear**	No	45	84.9	57.8	0.234	24.4	0.422	13.3	0.59
	Yes	8	15.1	87.5		37.5		25.0	

**9. Neck**	No	45	84.9	60.0	0.695	22.2	0.186	8.9	**0.013**
	Yes	8	15.1	75.0		50.0		50.0	

^*∗*^Several cell sizes are very small and have been excluded from final statistical analyses. The ones that are included in the interpretation of final results are described in bold text.

**Table 5:** Characteristics of chronic pain.

		Chronic pain					Fisher's
Variable		No	Yes	Total	% no	% yes	*p* value
Gender	Female	10	4	14	71.4	28.6	0.748
	Male	25	14	39	64.1	35.9	

Staging	Stage 1 or 2	4	4	8	50.0	50.0	0.679
	Stage 3 or 4	17	9	26	65.4	34.6	

Surgery	No	22	10	32	68.8	31.3	0.768
	Yes	13	8	24	54.2	33.3	

Chemo	No	16	5	21	76.2	23.8	0.247
	Yes	19	13	32	59.4	40.6	

Opioids	No	18	2	20	90.0	10.0	**0.006**
	Yes	17	16	33	51.5	48.5	

Gabapentin	No	33	12	45	73.3	26.7	0.014
	Yes	2	6	8	25.0	75.0	

TCA	No	32	15	47	68.1	31.9	0.397
	Yes	3	3	6	50.0	50.0	

NSAID	No	28	11	39	71.8	28.2	0.191
	Yes	7	7	14	50.0	50.0	

Number of sites of pain	0	20	1	21	95.2	4.8	**<0.0001**
	1	11	4	15	73.3	26.7	
	2+	4	13	17	23.5	76.5	

Variable	Chronic pain	N	Mean	Std. dev.	Std. error	*t*-test	
Age	Pain = no	35	62.23	13.831	2.338	0.425	
	Pain = yes	18	59.28	9.916	2.337		

Dose	Pain = no	35	5854	1562.68	264.14	0.149	
	Pain = yes	18	6470	1191.5	280.84		

Duration	Pain = no	35	42.37	14.534	2.457	0.604	
	Pain = yes	18	44.33	8.957	2.111		

**Table 6:** Surgery and medications.

Surgery	No	Yes	Total	Fisher's *p* value
	*Use of opioids*			
No	14	18	32	0.3859
Yes	6	15	21	

	*Use of gabapentin*			
No	29	3	32	0.2403
Yes	16	5	21	

	*Use of TCA*			
No	28	4	32	1
Yes	19	2	21	

	*Use of NSAIDs*			
No	23	9	32	1
Yes	16	5	21	

**Table 7:** Chemotherapy and medications.

Chemo	No	Yes	Total	Fisher's *p* value
	*Use of opioids*			
No	9	12	21	0.5733
Yes	11	21	32	

	*Use of gabapentin*			
No	16	5	21	0.2403
Yes	29	3	32	

	*Use of TCA*			
No	18	3	21	0.6711
Yes	29	3	32	

	*Use of NSAIDs*			
No	13	8	21	0.2017
Yes	26	6	32	

**Table 8:** XRT dose and medications.

		*n*	Mean	Std. dev.	Std. error	*p* value	Test
Opioids	No	20	5398.00	1910.38	427.17	0.05	Mann–Whitney
	Yes	33	6466.58	937.33	163.17		

Gabapentin	No	45	5981.80	1561.94	232.84	0.342	*t*-test
	Yes	8	6522.00	579.23	204.79		

TCA	No	47	6101.21	1422.24	207.45	0.603	*t*-test
	Yes	6	5766.67	1899.12	775.31		

NSAID	No	39	5922.69	1618.93	259.24	0.247	*t*-test
	Yes	14	6455.14	839.43	224.35		

**Table 9:** XRT duration and medications.

		*n*	Mean	Std. dev.	Std. error	*p* value	Test
Opioids	No	20	38.20	16.13	3.61	0.277	Mann–Whitney
	Yes	33	45.97	9.51	1.66		

Gabapentin	No	45	41.89	13.36	1.99	0.124	*t*-test
	Yes	8	49.50	6.97	2.46		

TCA	No	47	42.81	11.92	1.74	0.72	*t*-test
	Yes	6	44.83	20.19	8.24		

NSAID	No	39	42.56	13.42	2.15	0.659	*t*-test
	Yes	14	44.36	11.47	3.07		

## Data Availability

The clinical data used to support the findings of the study are restricted by the Springfield Committee for Research in Human Subjects (SCRIHS) in order to protect patient privacy. Data are available from Krishna Rao for researchers who meet the criteria for access to confidential data.

## References

[1] Woolf C. J., Mannion R. J. (1999). Neuropathic pain: aetiology, symptoms, mechanisms, and management. *The Lancet*.

[2] Ceyhan D., Gulec M. S. (2010). Is postoperative pain only a nociceptive pain?. *Journal of the Turkish Society of Algology*.

[3] Park H. J. (2014). Chemotherapy induced peripheral neuropathic pain. *Korean Journal of Anesthesiology*.

[4] Kumar S. (2011). Cancer pain: a critical review of mechanism-based classification and physical therapy management in palliative care. *Indian Journal of Palliative Care*.

[5] Roland N. J., Paleri V. (2011). *Head and Neck Cancer: Multidisciplinary Management Guidelines*.

[6] Kawecki A., Krajewski R. (2014). Follow-up in patients treated for head and neck cancer. *Magazine of European Medical Oncology*.

[7] Denaro N., Merlano M. C., Russi E. G. (2016). Follow-up in head and neck cancer: do more does it mean do better? A systematic review and our proposal based on our experience. *Clinical and Experimental Otorhinolaryngology*.

[8] British Association of Head and Neck Oncologists (2001). Practice care guidance for clinicians participating in the management of head and neck cancer patients in the UK. Drawn up by a Consensus Group of Practising Clinicians. *European Journal of Surgical Oncology*.

[9] Merskey H., Bogduk N. (1994). *Classification of Chronic Pain*.

[10] Mirabile A., Airoldi M., Ripamonti C. (2016). Pain management in head and neck cancer patients undergoing chemo-radiotherapy: clinical practical recommendations. *Critical Reviews in Oncology/Hematology*.

[11] Epstein J. B., Stewart K. H. (1993). Radiation therapy and pain in patients with head and neck cancer. *European Journal of Cancer Part B: Oral Oncology*.

[12] Costigan M., Scholz J., Woolf C. J. (2009). Neuropathic pain: a maladaptive response of the nervous system to damage. *Annual Review of Neuroscience*.

[13] Kehlet H., Jensen T. S., Woolf C. J. (2006). Persistent postsurgical pain: risk factors and prevention. *The Lancet*.

[14] Christianson C. A., Corr M., Firestein G. S., Mobargha A., Yaksh T. L., Svensson C. I. (2010). Characterization of the acute and persistent pain state present in K/BxN serum transfer arthritis. *Pain*.

[15] Feizerfan A., Sheh G. (2015). Transition from acute to chronic pain. *Continuing Education in Anaesthesia Critical Care & Pain*.

[16] Kuo P.-Y., Williams J. E. (2012). Pain control in head and neck cancer. *Head and Neck Cancer*.

[17] Srivastava P., Kingsley P. A., Srivastava H., Sachdeva J., Kaur P. (2015). Persistent post-radiotherapy pain and locoregional recurrence in head and neck cancer-is there a hidden link?. *Korean Journal of Pain*.

[18] Fink R. (2000). Pain assessment: the cornerstone to optimal pain management. *Baylor University Medical Center Proceedings*.

[19] Ranpura V., Agrawal S., Chokshi P. (2015). Improving documentation of pain management at MedStar Washington cancer Institute. *Journal of Oncology Practice*.

[20] Liu X., Gao X.-L., Liang X.-H., Tang Y.-L. (2016). The etiologic spectrum of head and neck squamous cell carcinoma in young patients. *Oncotarget*.

[21] Larizadeh M. H., Damghani M. A., Shabani M. (2014). Epidemiological characteristics of head and neck cancers in southeast of Iran. *Iranian Journal of Cancer Prevention*.

[22] The Demographic Statistical Atlas of the United States-Statistical Atlas, https://statisticalatlas.com/state/Illinois/Overview

[23] Rettig E. M., D’Souza G. (2015). Epidemiology of head and neck cancer. *Surgical Oncology Clinics of North America*.

[24] Silvestri F., Bussani B., Stanta G., Cosatti C., Ferlito F. (1992). Supraglottic versus glottic laryngeal cancer: epidemiological and pathological aspects. *ORL*.

[25] Epstein J. B., Wilkie D. J., Fischer D. J., Kim Y.-O., Villines D. (2009). Neuropathic and nociceptive pain in head and neck cancer patients receiving radiation therapy. *Head & Neck Oncology*.

[26] Ad V. B., Weinstein G., Dutta P. R. (2010). Gabapentin for the treatment of pain syndrome related to radiation-induced mucositis in patients with head and neck cancer treated with concurrent chemoradiotherapy. *Cancer*.

[27] Gomes T., Juurlink D. N., Antoniou T., Mamdani M. M., Paterson J. M., van den Brink W. (2017). Gabapentin, opioids, and the risk of opioid-related death: a population-based nested case-control study. *PLoS Medicine*.

[28] Murphy B. A., Beaumont J. L., Isitt J. (2009). Mucositis-related morbidity and resource utilization in head and neck cancer patients receiving radiation therapy with or without chemotherapy. *Journal of Pain and Symptom Management*.

[29] Vistad I., Cvancarova M., Kristensen G. B. (2011). A study of chronic pelvic pain after radiotherapy in survivors of locally advanced cervical cancer. *Journal of Cancer Survivorship*.

[30] Poleshuck E. L., Katz J., Andrus C. H. (2006). Risk factors for chronic pain following breast cancer surgery: a prospective study. *Journal of Pain*.

[31] Peuckmann V., Ekholm O., Rasmussen N. K. (2009). Chronic pain and other sequelae in long-term breast cancer survivors: nationwide survey in Denmark. *European Journal of Pain*.

[32] Cooper J. S., Fu K., Marks J., Silverman S. (1995). Late effects of radiation therapy in the head and neck region. *International Journal of Radiation Oncology∗Biology∗Physics*.

[33] Portenoy R. K., Lesage P. (1999). Management of cancer pain. *The Lancet*.

[34] Glastonbury C. M., Parker E. E., Hoang J. K. (2010). The postradiation neck: evaluating response to treatment and recognizing complications. *American Journal of Roentgenology*.

[35] Deng J., Ridner S. H., Dietrich M. S. (2012). Prevalence of secondary lymphedema in patients with head and neck cancer. *Journal of Pain and Symptom Management*.

[36] Delanian S., Lefaix J.-L. (2004). The radiation-induced fibroatrophic process: therapeutic perspective via the antioxidant pathway. *Radiotherapy and Oncology*.

[37] Bach C.-A., Wagner I., Lachiver X., Baujat B., Chabolle F. (2012). Botulinum toxin in the treatment of post-radiosurgical neck contracture in head and neck cancer: a novel approach. *European Annals of Otorhinolaryngology, Head and Neck Diseases*.

[38] Daele D. J. V., Finnegan E. M., Rodnitzky R. L. (2002). Spasm after radiotherapy: management with botulinum toxin a injection. *Archives of Otolaryngology–Head & Neck Surgery*.

